# The Role of Reduced Graphene Oxide toward the Self-Assembly of Lignin-Based Biocomposites Fabricated from Ionic Liquids

**DOI:** 10.3390/ijms19113518

**Published:** 2018-11-08

**Authors:** Dalia Al-shahrani, Stacy A. Love, David Salas-de la Cruz

**Affiliations:** 1 Department of Chemistry, Rutgers—The State University of New Jersey, Camden, NJ 08102, USA; alshahrani.dalia@gmail.com; 2 Center for Integrative and Computational Biology, Rutgers—The State University of New Jersey, Camden, NJ 08102, USA; asbff20@gmail.com

**Keywords:** lignin, reduced graphene oxide (rGO), ionic liquids, biocomposites, morphology, xylan

## Abstract

Lignin’s immiscibility with most polymers along with its unknown association behaviors are major factors that contribute to its disposal and processability for the production of materials. To fully utilize lignin, an improved understanding of its interaction with other materials is needed. In this study, we investigate the morphological and physicochemical properties upon the addition of reduced graphene oxide (rGO) as a function of material composition in a tertiary system comprised of lignin, cellulose and xylan. The main motivation for this work is to understand how the lignin molecule associates and behaves in the presence of other natural macromolecules, as well as with the addition of reduced graphene oxide. The fabricated biocomposites with and without rGO were investigated using Attenuated Total Reflectance Fourier Transform Infrared spectroscopy (ATR-FTIR), Scanning Electron Microscope (SEM) techniques, Thermogravimetric Analysis (TGA), and Differential Scanning Calorimetry (DSC). The results demonstrated that the regenerated films’ structural, morphological and thermal character changed as a function of lignin-xylan concentration and upon the addition of rGO. We also observed a dramatic change in the glass transition temperature and topography. Final analysis showed that the addition of rGO prevented the macromolecules to self-assemble through a reduction of π-π aggregations and changes in the cellulose crystallinity.

## 1. Introduction

The use of biologically renewable biomass with unique physical and functional properties is emerging as an important aspect of economic development, as well as manufacturing opportunities that could minimize the consumption of energy and the generation of waste [1]. Lignocellulosic biomass is an important feedstock for the use of renewable resources in emerging applications for packaging, production of fuels, materials, chemicals, and energy [2]. About 75% of lignocellulosic biomass components consist of polysaccharides which are present in the cell walls of woody plants [3]. Lignocellulosic biomass consists of three major macromolecules: lignin, cellulose, and hemicellulose [3]. In the natural structure of the lignocellulosic material, cellulose is embedded in a matrix of hemicelluloses, and the network between the cellulose-hemicellulose is further encapsulated by an outer lignin layer [4]. This network occurs mostly through extensive hydrogen bonding between all three components [5]. There is also covalent bonding among the components, such as an ester bond between lignin and hemicellulose, and ether bonds between cellulose and lignin [6,7]. Lignin is a component that fills the spaces between hemicellulose and cellulose, where it acts as a resin that holds the lignocellulosic matrix together [8]. These natural interactions enable the formation of hierarchical fibers for which its properties depend on material structural complexity, molecular distribution and composition. For the production of materials, lignin is not usually utilized and it is discarded away. 

Lignin is a three-dimensional amorphous polymer consisting of aromatic methoxy phenyl-propane structures. As a natural polymer with an irregular structure and cross-linked chains, we should understand both the structure and function of lignin [9]. Lignin consists of three monolignol monomers as its building blocks: *p*-coumaryl, coniferyl and sinapyl alcohols. These alcohol monomers are categorized by the number of methoxy groups on their phenolic ring [10]. The molecular weight, composition and amount of lignin monolignol differ from plant to plant. For example, in softwood, the monolignols coniferyl represent 90% of the structure, while equal proportions of coniferyl and sinapyl are found in hardwood [11]. Due to this heterogenous structure and linkages, it is still a major challenge to convert lignin into a valuable material [12]. This also could be related to the presence or absence of the inter or intra-molecular interaction between lignin and other carbohydrate components and due to lignin high molecular weight [13]. As a result, it is particularly difficult to dissolve lignin in common solvent systems, this causes aggregation and miscibility problems resulting in phase separation, even in commercially processed lignin with low molecular weight. The aggregation (self-assembly) is governed by the non-bonded orbital π–π interaction among the aromatic groups and van der Waals attractions [14]. To increase the usefulness of lignin in material applications, we have to understand the lignin structure and it’s self-assembly in a multicomponent system. Preventing its self-assembly will provide a path to utilize it in the production of fibers, aerogel and films for medical textile [15], car bumper manufacturing [16], insulation [17] and bio-batteries [17,18].

Lignin has hydroxyl and ether groups available for hydrogen bonding, which could help to increase the intermolecular interaction between its matrix by disrupting the hydroxyl groups in the backbone and preventing these bonds from wrapping around themselves [19]. Disrupting those intermolecular interaction bonds will increase the usability of lignin’s structure. Due to the availability of the lignin’s linkages, we can blend it with other natural and compatible polysaccharides such as cellulose. Cellulose is an essential biopolymer found in the cell walls of plants. It is considered to be the most abundant material in the world and is found in nature as cellulose I (Iα or Iβ) [20]. Cellulose is a linear homo-polysaccharide composed of repeating (β1-4) d-glucose units and is stabilized by van der Waals forces and hydrogen bonds between hydroxyl groups and oxygens of adjacent molecules. It is capable of forming strong intermolecular and intramolecular hydrogen bonds via its β(1-4) glycosidic bonds, further providing possible avenues for enhancing its molecular interactions with lignin [21]. In this project, cellulose will be used as an adhesion agent. Cellulose contains both crystalline and amorphous regions and with its extensive hydrogen network, its interaction with lignin can be either hindered or enhanced. To solve this problem, a component of hemicellulose, xylan, will be added to the system to control the cellulose adhesion capabilities. In the xylan backbone, the glucan of 1,4-d-xylopyranose holds branched chains of 4-oxymethylglucuronic acid and has a strong interaction with cellulose [21]. Xylan could add levels of disruption to the cellulose crystalline structures, especially in the formation of microfibrils [22,23].

The extensive network of inter- and intra-molecular hydrogen and dispersive bonds of the lignocellulosic biomass could impede its dissolution in most solvents. To dissolve all of the lignocellulosic mixture, we used ionic liquids [24]. Ionic liquids (IL) are one of the most popular solvents for natural polymers and have been shown to dissolve natural macromolecules for film synthesis without breaking its molecular weight. ILs have unique properties, such as low vapor pressure, high thermal and mechanical stability, low toxicity, and non-flammability [10]. The ionic liquid will dissolve the original materials (in our case, cellulose, lignin and xylan). The dissolution proceeds through a weakening of the extensive inter/intramolecular hydrogen bonding network of the macromolecules. Studies have shown that, specifically, this occurs due to relatively strong interactions between the ionic liquid’s anion and the hydrogen bond-accepting functional groups of the biopolymers [25]. After the dissolution, water is added to wash away the IL. During this phase, the anions and cations migrate from the biopolymer blend to the water, resulting in the formation of the solid biocomposites [26]. In this investigation, 1-allyl-3-methylimidazolium chloride (AMIMCl) will be used as a solvent which has been show to dissolve cellulose, lignin and xylan [27]. 

In previous studies, we noticed that when we increased the content of lignin within lignocellulosic films fabricated from ILs, the lignin tended to wrap around itself [8]. Thus, to prevent the self-assembly of lignin as a function of polysaccharide content, a spacer material must be added to the blend. Graphene oxide (GO) is an ideal compound to increase the interaction and stabilization of lignocellulose components, especially when its surface has been reduced with hydroxide groups [28]. GO is made of modified graphene sheets with a number of oxygen functional groups, such as hydroxyl, epoxy, carbonyl and carboxyl [28]. GO exhibits mechanical strength, conductivity and a highly specific area. Its sheets possess a high adsorption capacity and have good solubility due to their abundant surface oxygen-containing groups. These groups provide sufficient sites for linking other macromolecules and polymers, thus enhancing physicochemical properties [29,30]. It was found recently that lignin can easily bind with reduced graphene oxide (rGO) sheets by hydrogen bonding and π–π stacking [28,31,32]. The large surface area of rGO makes it an ideal conducting matrix for trapping lignocellulosic components, especially lignin, between its two-dimensional sheets [31]. In addition, IL increases the space between π–π aggregation of both rGO and lignin’s aromatic rings, which could prevent self-assembly and enhance blending capabilities [14]. To that end, reduced graphene oxide (rGO) was used as an intermediator to immobilize the lignocellulosic biomass between its two-dimensional flat sheets and to prevent the macromolecules in the blend to self-assemble.

To utilize cellulose, lignin, xylan and rGO in different kinds of applications, it is essential to compose stable homogenous films in order to effectively modify its properties. Changing the ratio of the lignocellulosic biofilms with the addition of rGO can result in structural modifications that affect the thermal stability and topography of these films. However, the characterizations of blended structures based on lignocellulosic biomass (lignin, cellulose, xylan) films with rGO in various ratios, fabricated from AMIMCl and coagulated with water, have not yet been fully explored. Our investigation focuses on comparing the physicochemical properties of each blend of lignocellulosic biomaterial individually and with rGO. Several characterization techniques such as ATR-FTIR, SEM, TGA and DSC were used to elude on the material properties changes. The results and subsequent analyses were used to explain the changes in the structure and morphology of the blended films and the profound morphological and thermal changes with the introduction of rGO into the matrix. 

## 2. Results and Discussion

### 2.1. Structural, Thermal and Morphological Changes

Multiple macromolecular compositions were chosen to test if the rGO would prevent the molecular self-assembly of lignin. Table 1 shows the composition breakdown of lignin, xylan and cellulose. The total weight of the biocomposites accounts for 10% *w*/*w* and the ionic liquid accounted for 90% *w*/*w* of the total weight. The content of rGO is 1% of the total weight of the biocomposites (not including the ionic liquid). Our working hypothesis states that in a blended system composed of cellulose, xylan and lignin, the fibrous assembly will be impacted by material concentration. Also, we hypothesized that the fibrous assembly will be disrupted upon the addition of rGO. Furthermore, we want to determine the extent to which the molecular interactions will affect the biofilm’s morphological and thermal properties. 

**Figure 1:**
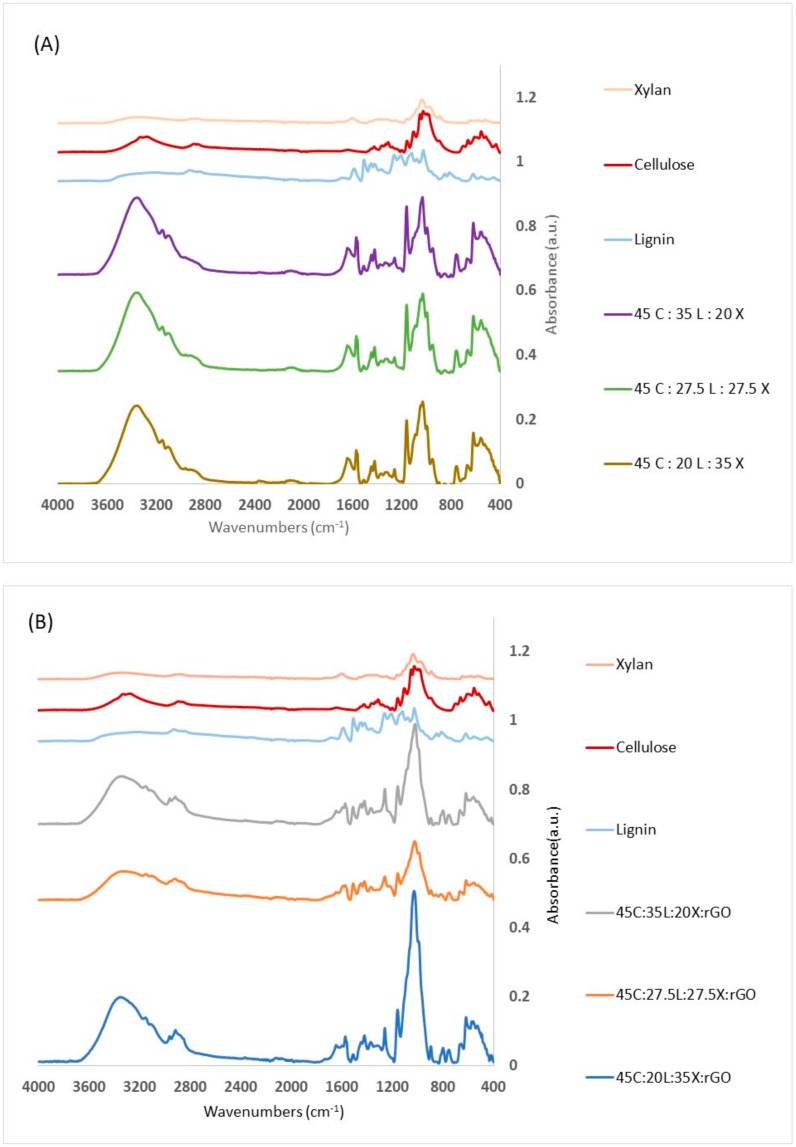
FTIR spectra of (**A**) material components of lignocellulosic films without rGO and (**B**) of material components of lignocellulosic films with rGO.

The FTIR results show the absorbance spectra between the pure components of the lignocellulosic materials and lignin-based biomaterials with and without rGO (Figure 1). The IR spectra within the broad regions of OH (3200 cm^−1^) and C–O (around 1060 cm^−1^) were assigned to cellulose. Xylan has a stronger peak at 1600 cm^−1^ which is indicative of C=O stretching. On the other hand, lignin shows the most unique IR expression in the fingerprint region (700–1800 cm^−1^) with especially strong peaks at the methoxy (1450 cm^−1^), aromatic (1400–1600 cm^−1^) and carbonyl (1670–1820 cm^−1^) groups. Other bands occurring from 1050 to 1150 cm^−1^ are characteristic of C–O [1]. The major IR peaks for AMIMCl are attributed to 3050, 2965, 1570, and 1165 cm^−1^, and have been previously characterized by others [33]. The IR spectrum of a pure graphene sample (not shown here) shows O–H (3200–3700 cm^−1^), C=O (1670–1820 cm^−1^), C–O–C (1200 cm^−1^) and C–O (1050–1150 cm^−1^). Some of these regions are attributed to epoxy ring deformation and C–O stretching mixed with C–O–C bending. 

As can be seen from the IR for the lignin-based biomaterials with and without rGO, the spectra of the blended films correspond to the predominant constituent, lignocellulosic biomass and rGO. Holding the cellulose concentration steady allows us to see if the changes observed are due to the alternation between xylan and lignin. In Figure 1A, the blended films of lignin-based biomaterials have more absorbance intensities compared to the pure component. Each biocomposite shows the existence of each of the pure materials. We can notice the OH (3200 cm^−1^) and C–O (around 1060 cm^−1^) arising from cellulose and C=O stretching from Xylan. In addition, we can observe lignin fingerprint regions (700–1800 cm^−1^) with especially strong peaks at the methoxy and carbonyl groups. This enables us to conclude that all materials are located within the matrix of the film. We can also observe a band at 1165 cm^−1^ in all samples. This is indicative of AMIMCl which may be trapped between chains in the structures of lignocellulosic blended films. We suspect that less than 0.5% of IL are trapped in this matrix. This can be explained by the hydrogen bonds that occur between hydroxyl groups in the lignocellulosic materials and the cation (imidazolium ring) or anion (Cl^−^) groups of the AMIMCl.

In Figure 1B, the lignocellulosic biomass with rGO blended films shows similar band regions as the films without rGO. We can notice a broad –OH band interacting across the blend with a shift to a higher wavenumber when the lignin concentration increases. The lignocellulosic blended film with rGO gives a complex of –OH and C–O peaks between 900–1600 cm^−1^ which are due to the C–O–H and C=O of the COOH groups [28]. We also observed that biocomposites with rGO have less ionic liquid retention during coagulation. This study provides evidence that each of the components are present and that they are interacting within the films. 

### 2.2. Morphology Analysis (SEM)

**Figure 2:**
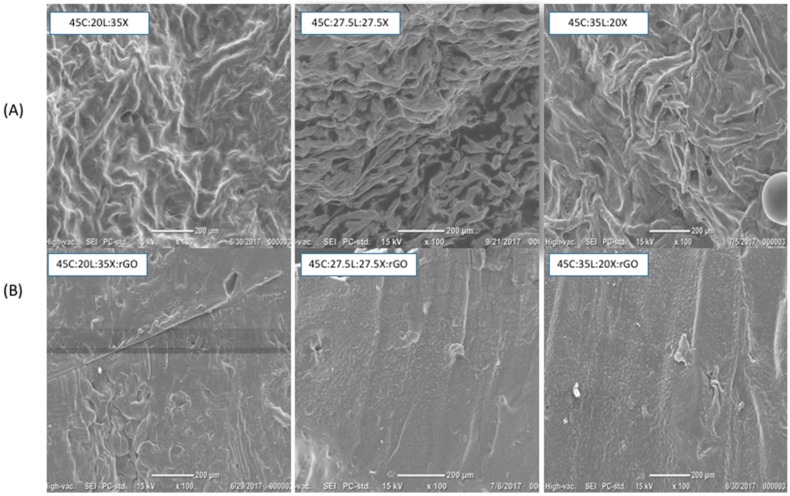
SEM images: (**A**) lignocellulosic blended films and (**B**) lignocellulosic blended films with rGO.

As can be seen in Figure 2, the SEM images illustrate marked changes in film morphology according to alternate lignin-xylan proportions in the blended biopolymer film with and without rGO. The morphology and structure of the lignocellulosic blended films without rGO are shown in Figure 2A. The lignocellulosic blended films with less lignin (20% and 27.5%) and more xylan content show a fibrous pattern on their surface. As the lignin content increases, the fiber pattern formation is enhanced. This provides evidence that demonstrates the capabilities of lignin to wrap around the other two macromolecules emulating natural phenomena. The lignocellulosic blended films with rGO are shown in Figure 2B. The addition of rGO to the blended films results in smoother surfaces compared to the films with only lignocellulosic blends. The rGO and lignocellulosic blends interact via intermolecular hydrogen bonding, preventing the material to self-assemble [28]. It is suggested that rGO prevents lignin from self-assembling through trapping the lignin between its 2D sheets and providing molecular interactions via rGO’ s hydrogen bonds within the lignocellulosic blends. This is a very important implication, as it can provide evidence that the rGO prevents the macromolecule self-assembly in the tertiary system of the lignin-based composites and further suggests that its topological structure is independent of lignin concentration. 

### 2.3. Thermal Decomposition Analysis

**Figure 3:**
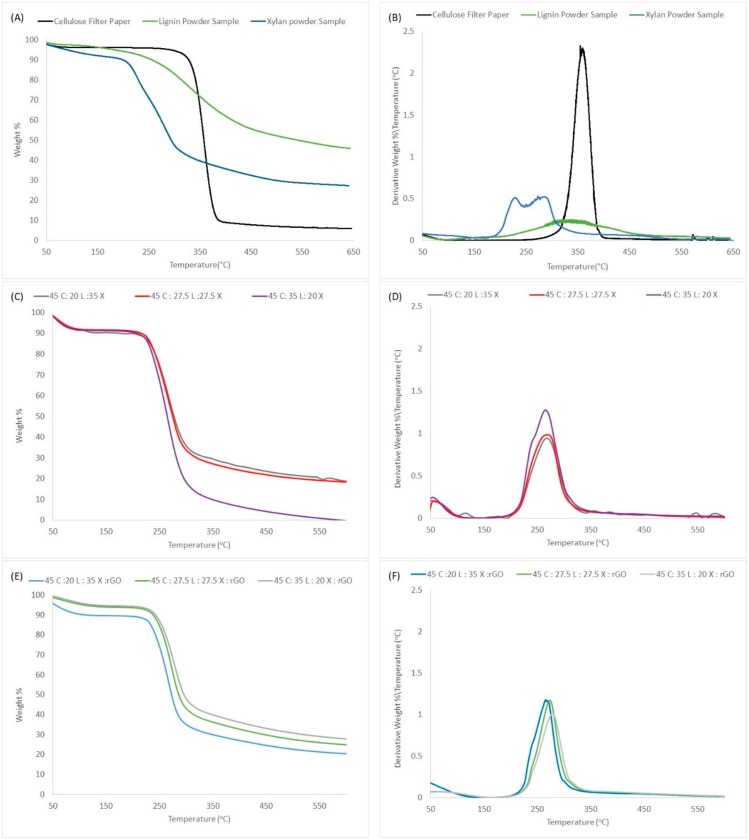
TGA percent weight decomposition analysis for (**A**) pure Samples, (**C**) lignocellulosic blended films and (**E**) lignocellulosic blended films with rGO. TGA derivative percent weight decomposition analysis for (**B**) pure samples, (**D**) lignocellulosic blended films and (**F**) lignocellulosic blended films with rGO.

The thermal analysis curves show the structural modifications effect on thermal stability as measured by decomposition temperatures. In Figure 3, TGA thermograms represent the pure natural materials and the resulting lignocellulosic blended films with and without rGO. Table 2 show the overall quantitative data. At first glance, we can observe a one-step decomposition for all biocomposites including the film regenerated with rGO. For comparison, we added the curves for the pure samples (cellulose, xylan and lignin). The curves show that xylan has a lower decomposition temperature with a two-step transition followed by cellulose and lignin with one-step transitions. As for lignin, pure lignin degrades slowly over a larger span starting at 250 °C. This characteristic can be seen in the widening of the derivative peak in Figure 3B. This data is important because it enables us to conclude that the biocomposites are homogeneous (that is, all composites are well integrated in the overall structure of the film which results in a one-step decomposition).

In continuation with the analysis, we can observe a slight shoulder at 240 °C in the derivate curves for all biocomposites. This peak seems to be affected by changes in the concentration of xylan. Also, we know that xylan molecules are going to degrade before lignin and cellulose. As a result, we can suggest that his shoulder is related partially to the decomposition of xylan in the sample. Also, we can observe that the *T* onset is independent of concentration. Upon the addition of rGO, *T* onset increases 10 °C, see Table 2. Interestingly, when the rGO was added, the overall weight lost percentage became more stable; however, it slightly increases as a function of lignin content. In addition, we noticed that for the film 45C:35L:20X:rGO the *T*_max_ increases 14 °C and weight loss is reduced from 97.75% to 70.76% as compared with film with the same concentration but without rGO. Therefore, we can make several assumption, (i) thermal degradation is dependent on lignin content; (ii) the rGO is well integrated within the matrix, causing it to resist additional thermal stress and (iii) the rGO prevents drastic thermal changes as a function of material composition. 

### 2.4. Glass Transition Temperature

**Figure 4:**
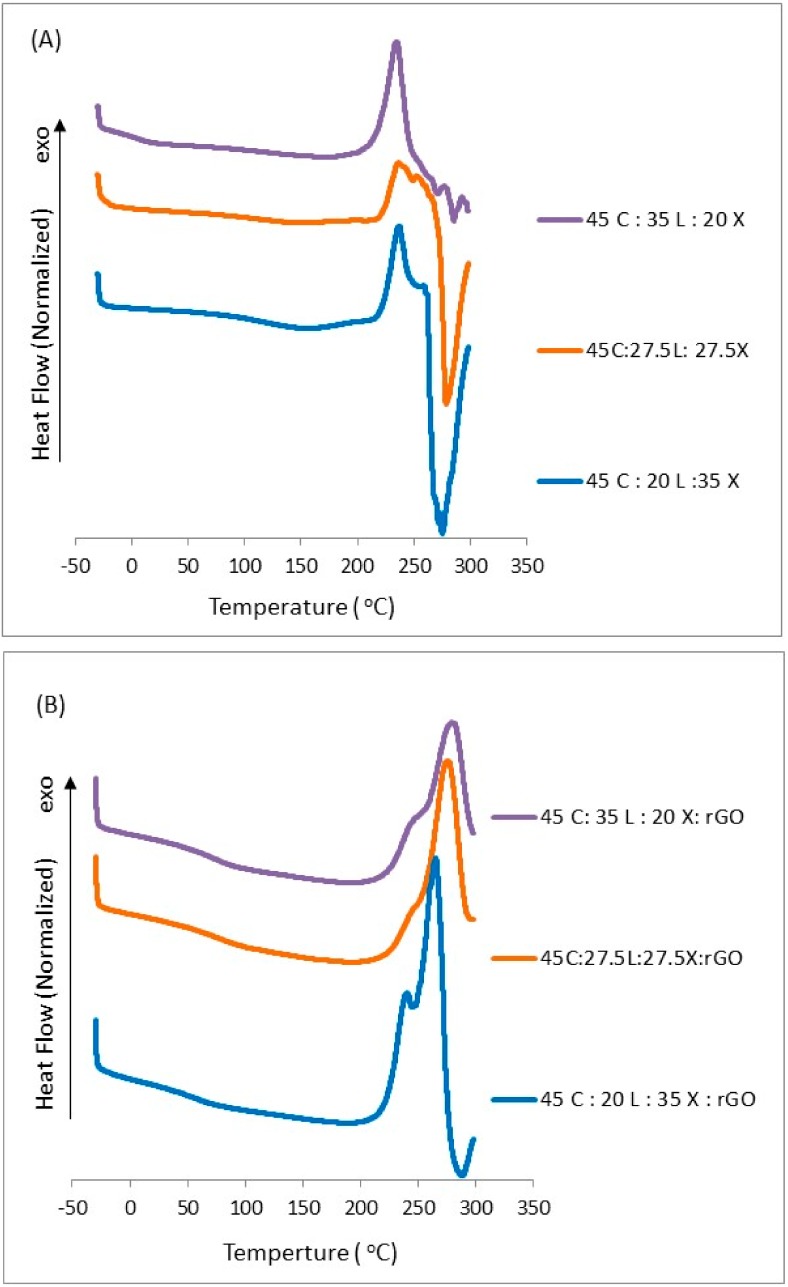
DSC curves of blended lignocellulosic films (**A**) without rGO and (**B**) with rGO.

Thermal transition studies on lignocellulosic biomass films with and without rGO were carried out through Differential Scanning Calorimetry (DSC). Figure 4 shows the thermograms for all biocomposites without and with rGO. The films without rGO show mostly three transitions: glass transition (*T*_g_), crystallization and meting. The glass transition occurred at 120 °C and it is independent of concentration. As the lignin content decreases, the maximum crystallization temperature (*T*_c_) changes from 239, 239, and 236 °C. This exothermic transition upon heating has been observed in other films composed of natural components [26,34]. In our biocomposite matrix, xylan molecules started to degrade first (see TGA thermograms). This led the cellulose backbone chains to crystallize before decomposition. As we can notice, this exothermic transition is followed by an endothermic transition as a result of melting, which is quickly followed by its degradation. We do not expect xylan nor the lignin structure to melt as they do not form crystalline domains. This melt transition is higher for the 45C:20L:35X films. We suspect that this biocomposite, which has a higher composition of xylan or lower composition of lignin, contains amorphous regions which allow the cellulose backbone chains to quickly crystallize and melt. Since the lignin content is low, overall interactions within the matrix will be dominated by the xylan 4-oxymethylglucuronic acid group which can interact with the d-glucose units of the cellulose. This interaction alters the formation of crystal domains at higher temperatures by lessening the ability of lignin’s coniferyl aldehyde and phenolic groups to interact within the cellulose matrix. In fact, it is known that xylan can disrupt the overall cellulose crystalline structure. This could explain the reason for why this film showed lower fibrous-like patterns in the SEM section. Upon the addition of rGO, we noticed that all three samples exhibit mostly two transitions: *T*_g_ and *T*_c_. The *T*_g_ for these films were reduced to 85 °C, a difference of 35 °C compared to the films without rGO. Surprisingly, the glass transition temperature is independent of the material concentration, similar to the film without rGO. As the lignin content decreases, the maximum crystallization temperature is reduced (281, 276 and 266 °C). The maximum crystallization temperature shows a 42 °C difference as compared with the film without rGO. Furthermore, we can notice that the endothermic transition disappeared upon the addition of rGO into the system. This indicates that the rGO contributed to strong intermolecular hydrogen bonds with the macromolecules which prevents the melting just before the maximum point of degradation. We can now suggest from our findings that the lignin content has a definitive effect on crystallinity. This crystallization is not only dependent on lignin content but also on rGO content which can affect thermal stability. This suggests that more energy is required to break down the bonds in these lignocellulosic films, yielding a more stable material. DSC and TGA analysis results provide evidence to suggest that the enhancement in the thermal characteristics of lignocellulosic films with rGO is the result of its good thermal conductivity and due to its molecular interaction with the macromolecules which directly affect the film morphology.

To summarize, we can conclude from the various characterization analyses that all molecular structures including rGO were embedded into the matrix successfully. The IR spectra for the lignocellulosic blended films indicated that adding rGO increased the intermolecular interaction within the blended films. The topological morphology of the films without rGO has small fibrous patterns; however, upon the addition of rGO, such fibrous patterns disappeared. The TGA results showed that the thermal stability is dependent on lignin content. Also, it uncovered that the thermal stability improved upon the addition of rGO. Furthermore, adding rGO resulted in a reduction in the glass transition temperature, causing changes in the cellulose crystallization and melting transitions. These results confirm the natural state of lignin’s ability to tune the thermal stability through its abundance of aromatic and non-polar species and the ability of rGO to imbed itself within the matrix.

## 3. Materials and Methods

### 3.1. General Materials

Lignin alkali (CAS: 8068-05-1) was purchased from Sigma Aldrich (St. Louis, MO, USA) while the Whatman Grade Number 1 Cellulose Filter Paper was purchased through Fischer Scientific (Pittsburgh, PA, USA). Xylan was obtained from beechwood (CAS:9014-63-5) and the dispersed graphene oxide (GO) in solution was purchased from Aldrich (St. Louis, MO, USA) (Product #763705). Ionic liquid 1-allyl-3-methylimidazolium chloride (AMIMCl) was purchased from Alfa Aesar (Tewksbury, MA, USA) (Product #H26952). The various characterization results shown in this paper demonstrate the properties of these materials. 

### 3.2. Fabrication Method of Biocomposite

The regenerated biomass films were prepared by using a modified version of the protocol seen in previous literature [8,32]. To increase the compatibility of the GO sheets with lignin and to increase its adhesion capabilities, GO was reduced with 0.005 g Vitamin C in 1 mL water and then heated to 50 °C for 24 h. After that, it was rinsed five times with water in a centrifugation process to remove the remaining Vitamin C. After which, the rGO was filtrated by pouring it through cellulose filter paper and then dried for 24 h. The AMIMCl was placed in a test tube (approximately 90 *w*/*w*) and heated in oil at 90 °C, at which point the biomass components (lignin, xylan and cellulose paper with rGO approximately) were added to top 11 *w*/*w*. The rGO accounted for 1% of the total weight of the biocomposites. The biomass and ionic liquid solution was transferred and spread between two glass plates and fully submerged in distilled water for 24 h for IL removal and regeneration process. Once this was completed, the films were placed in a vacuum oven at 50 °C for 24 h for drying and then were later stored in a vacuum desiccator prior to testing.

### 3.3. Fourier Transform Infrared Spectroscopy 

Attenuated Total Reflectance Fourier Transform Infrared spectroscopy (ATR-FTIR) analysis was performed using a Bruker ALPHA-Platinum FTIR Spectrometer (Billerica, MA, USA) with Platinum-Diamond sample module and Bruker OPUS software version 7.2, Build: 7.2.139.1294. The spectra were collected in the wavenumber between 4000–400 cm^−1^, resolution 4 cm^−1^, 32 sample scans and 128 background scans. Six different locations on the film were analyzed for each sample and the spectra average was taken. 

### 3.4. Thermogravimetric Analysis 

Thermal data were obtained using Thermogravimetric Analysis (TGA), and were carried out on all samples in a nitrogen atmosphere on a TA Instruments Discovery system. We used the TA Discovery software to determine the derivative plot using peak height analysis. Blended film samples averaging 6 mg were heated linearly with a 10 °C/min ramp up to 650 °C. Step transition analyses were performed for each sample to determine the onset of decomposition (*T*_95%_) and the weight-loss percentage of the sample evolution during the main decomposition step. Derivative values were calculated, and peak height analyses were performed to determine the temperature at which the samples decomposed (*T*_ΔMax_).

### 3.5. Differential Scanning Calorimetry

Samples of approximately 5 mg weight were enclosed in TZero aluminum pans and run in a TA Instruments Discovery (New Castle, DE, USA) with a refrigerated cooling system (RCS) and nitrogen gas flow (50 mL min^−1^). The standard aluminum reference was used to calibrate the heat capacity. Samples were heated from 10 °C/min up to 150 °C to allow the water to evaporate. They were then cooled down to −30 °C and heated back up again to 300 °C at 10 °C/min. The glass transition temperature was computed by taking the middle point on the slope of shifting. 

### 3.6. Scanning Electron Microscope

The JEOL JCM-6000 Instrument (JEOL USA Inc, Padbody, MA, USA) was used to obtain the topological images for Scanning Electron Microscopy (SEM). A small piece of the blended film sample was mounted on carbon tape. A Denton Desk II Au-Pd sputter coater was used and the samples were sputter-coated in a light-vacuum of 10 V for 90 s. The samples were then placed into a desiccator for 24 h to ensure drying of the Au-Pd coating. Once the drying was complete, the samples were ready for SEM imaging. The magnification used in this paper is a scale bar of 100× (200,00 µm) at 15 kV.

## 4. Conclusions

Here, the structural and thermal properties of blended lignin, cellulose and xylan biocomposites fabricated from 1-allyl-3-methylimidazolium chloride ionic liquid with and without rGO were studied. We confirmed the ability of rGO in preventing molecular self-assembly and its ability in tuning thermal properties. Thus, we concluded that the utilization of rGO could be considered for the fabrication of medical textiles, insulation materials and bio-batteries using natural macromolecules such as lignin.

## Figures and Tables

**Table 1:** Experimental conditions of regenerated film concentrations of lignin, cellulose, xylan and rGO.

Cellulose (% wt)	Lignin (% wt)	Xylan (% wt)	rGO (% wt of Total Biocomposites)
45%	20%	35%	1
45%	27.5%	27.5%	1
45%	35%	20%	1

**Table 2:** TGA decomposition temperatures of blended films without and with rGO components and experimental conditions.

Without rGO	*T*_Onset_ (°C)	*T*_End_ (°C)	*T*_ΔMax_ (°C)	Wt Loss %
45C:20L:35X	245.448	299.907	267.393	79.60%
45C:27.5L:27.5X	245.209	300.687	270.227	80.84%
45C:35L:20X	244.286	297.369	264.747	97.75%
**With rGO**	***T*_Onset_ (°C)**	***T*_End_ (°C)**	***T*_ΔMax_ (°C)**	**Wt Loss %**
45C:20L:35X:rGO	256.595	289.231	264.921	77.72%
45C:27.5L:27.5X:rGO	254.047	299.955	272.569	72.98%
45C:35L:20X:rGO	254.470	305.045	278.00	70.76%
